# Infant Pupil Diameter Changes in Response to Others' Positive and Negative Emotions

**DOI:** 10.1371/journal.pone.0027132

**Published:** 2011-11-16

**Authors:** Elena Geangu, Petra Hauf, Rishi Bhardwaj, Wolfram Bentz

**Affiliations:** 1 Durham University, Psychology Department, Science Laboratories, Durham, United Kingdom; 2 Romanian Academy of Science, Department of Psychosocial Research, Cluj-Napoca, Romania; 3 St. Francis Xavier University, Department of Psychology, Antigonish, Nova Scotia, Canada; 4 Faculdade de Ciências da Universidade de Lisboa, Centro de Álgebra, Campo Grande, Lisboa, Portugal; French National Centre for Scientific Research, France

## Abstract

It has been suggested that infants resonate emotionally to others' positive and negative affect displays, and that these responses become stronger towards emotions with negative valence around the age of 12-months. In this study we measured 6- and 12-month-old infants' changes in pupil diameter when presented with the image and sound of peers experiencing happiness, distress and an emotionally neutral state. For all participants the perception of another's distress triggered larger pupil diameters. Perceiving other's happiness also induced larger pupil diameters but for shorter time intervals. Importantly, we also found evidence for an asymmetry in autonomous arousal towards positive versus negative emotional displays. Larger pupil sizes for another's distress compared to another's happiness were recorded shortly after stimulus onset for the older infants, and in a later time window for the 6-month-olds. These findings suggest that arousal responses for negative as well as for positive emotions are present in the second half of the first postnatal year. Importantly, an asymmetry with stronger responses for negative emotions seems to be already present at this age.

## Introduction

Infants are exposed from a very few days after birth to an increasingly complex social environment, in which other's emotions convey rich information about the ongoing interactions and the environment they encounter. Existent data suggests that infants are not only able to discriminate between different emotional expressions [1], [2] and successfully use this information to disambiguate the meaning of objects and situations [3], but that they also respond with matching affective states. Affect sharing is central to the ability to empathize with others. Empathy is an emotional response triggered by and isomorph with another's emotional state or condition, and which is modulated to a certain degree by regulatory mechanisms and awareness of the other as source of one's own affective state [4]. In the present study we aim at further understanding infants' affect sharing by investigating their emotional arousal responses to both positive and negative emotional expressions.

The affective arousal in response to others' emotions has been hypothesized to rely on different mechanisms. In one of them mimicry or perception-action coupling plays a central role, assuming that the perception of other's emotional expressions triggers the mimicry of that particular expression which, through feedback processes, leads to the activation of somatic and autonomic responses [5]–[7]. Alternatively, it has been hypothesized that affect sharing responses are simple emotional responses to conditioned or unconditioned emotional stimuli, which in this particular case are the emotional displays observed in others [8]–[10]. Although probably not mutually exclusive, current empirical evidence does not provide conclusive evidence in support of one over the other. This is even more true when considering their early development and how this relates to empathy development overall. For example, debate still exists whether and when infants are able to manifest mimicry of simple facial movements or emotional facial expressions (see [11] for a thorough review of the topic). But perhaps most importantly, there is still limited direct evidence for infants' emotional responses to others emotional displays [12]. Given the inconclusive evidence for whether affect sharing involves mimicry or whether they are simple emotional responses, we will further use the term emotional resonance to refer to any arousal responses triggered by exposure to others' affective displays.

As early as a few hours after birth, infants manifest similar emotional reactions in response to their peers' observed affect, by showing increased heart rate and crying face and voice to the sound of another infant cry [13]–[16]. This tendency to respond with matching emotional responses to other's negative affect persists throughout infancy [12], [17], [18], however, less is known about infants' emotional resonance to the positive emotions perceived in others. Limited direct evidence suggests that young infants respond emotionally to both the positive and the negative emotions observed in others. For example, when their own mother displays facial and vocal expressions of happiness, sadness, and anger, 2-month-old infants tend to respond significantly with matched facial expression categories [9]. Similar patterns of results have been recorded in 4- to 6- and 7- to 9-month-old infants, who responded with positive behaviours (i.e., smiles, approach body movements) to a static happy female face, and with negative behaviours (i.e., pre-cry face, avoidance movements of the body) to an angry face [19]. However, Vaish, Grossman, and Woodward [20] hypothesized that this similarity in matched affect responses between different emotions changes by the end of the first postnatal year, with a shift towards more emotional resonance to negative compared to positive emotions. Studies on how infants gather and receive emotional information from social referents have provided some indirect evidence in this respect. When an adult displays happy, fear and neutral facial and vocal cues towards an ambiguous object, 10- to 12-month-old infants show more negative affect in the fear than in the neutral condition, but not more positive affect in the happy compared to the neutral condition [21], [22].

Interestingly, a negativity bias in processing emotional expressions has received support from both adult and infant studies. Perceiving expressions of negative emotions (such as fear), but not positive emotions, is associated in adults with increased activation in brain areas specialized for processing emotional information and in areas relevant for action representation and production [23], [24]. In infants, enhanced allocation of attention and sensorial processing of negative emotional expressions emerges towards the end of the first year of life. At the age of seven months, the perception of angry voices is associated with larger negative ERP amplitude in frontal-central areas, suggesting increased attention for negative emotional expressions [25]. Later on, at the age of 12 months, angry faces lead to larger negative ERP amplitudes in the occipital area, suggesting increased sensorial processing [26]. When these negative emotional expressions are directed towards a specific stimulus in the environment, the information is further used in order to understand the situation and to generate the appropriate responses toward that object as seen in the 12-month-olds [21], [22], [27]. One possible explanation is that negative emotional expressions (face, voice, body) are more salient than the positive expressions; given that they convey information regarding possible threats in the environment relevant to either self or conspecifics [28].

This shift towards increased attention and sensorial processing of negative emotional information may also be relevant at the level of emotional resonance. In the current study we aimed to investigate the arousal responses to positive and negative emotions in 6- and 12-month-old infants. These ages are before and after the occurrence of the asymmetry in processing others' positive and negative emotions as suggested by previous studies [25], [26]. We expected therefore that older infants will manifest increased arousal towards negative compared to positive emotions expressed facially and vocally by peers. For younger infants, similar levels of arousal were expected to be recorded for positive and negative emotions.

Facial and vocal expressivity, along with other behavioural aspects, has been used to measure emotional resonance reactions in newborns and infants [9], [12], [16], [19]. However, subjectivity associated with coding and difficulties in generalizing the measurements across different age groups limit their use [29]. Several psychophysiological measurements have been proposed as valid measurements of emotional responses that can be used with infant populations as well (e.g., changes in heart activity, skin conductance, pupil dilation). Among these, pupil dilation is particularly relevant because it can be easily and reliably recorded without any additional distress for the infant participant due to technical procedures [30], [31].

The main function of the pupil is to regulate the amount of light entering the eye by fast changes in diameter [32]. These changes are driven by both sympathetic and parasympathetic branches of the autonomic nervous system, leading to dilation for low light conditions and constriction for bright environment or stimuli. Changes in pupil size also reflect emotional and cognitive responses [33]. First studies to suggest that pupil dilation is a sign of emotional arousal were conducted by Hess and Polt [34], who showed that when people view pleasant pictures, their pupil size increases. These changes in pupil size to emotionally loaded visual and auditory stimuli (e.g., the sound of a crying baby or people fighting, images of laughing people) occur concurrently with changes in the galvanic skin conductance response, while they are not related to attentional indexes, like the heart rated deceleration to the onset of the stimulation [35], [36]. Although specific cognitive processes are involved in processing the social information associated with this type of stimuli, there is evidence to suggest that the recorded autonomous arousal indexed by changes in pupil size is of sympathetic origin [37], [38]. Thus evidence is provided that pupil dilation is valid measure of limbic brain functioning and emotional arousal [39], [40]. Similar results have been obtained with children and infants. For example, when presented with pictures of their mother's face, 1- and 4-month-old infants manifest a larger pupil diameter compared to non-social stimuli like geometric checkerboard patterns [41]. Further, when children are required to identify the emotional valence of words, greater pupil dilation is shown to those with negative emotional load than to the neutral or positive ones [42]. Importantly, in both preschoolers and adults, observing situations that induce harm to others elicit an increase in pupil size [43].

In order to investigate whether valence influences 6- and 12-month-olds arousal responses, we recorded the changes in their pupil diameter during the presentation of video recordings of other infants displaying different emotions without cues for the initiation of social interaction. These allowed a more direct assessment of infants' emotional resonance reactions to other's emotions. We predicted that perceiving the positive and negative emotions of others induces larger pupil diameters when compared to the emotionally neutral states. Following the negativity bias hypothesis, we expected that 12- but not 6-month-old infants will show increased pupil diameters in response to the negative stimulus compared to the positive one. We also investigated the impact of the emotional expression perceived in others on the pattern of visual fixations of the stimulus. In particular we were interested whether infants preferentially process visually the socio-emotional relevant aspects of the stimulus (i.e., the face of their peer), and if this differs between conditions.

## Methods

The research reported in this manuscript has been conducted in accordance with the principles expressed in the Declaration of Helsinki. All parents signed an informed consent for their infants to participate in this study. The research complies also with the ethical principles advanced by the Research Ethics Board at St. Francis Xavier University, from which it received approval.

### *Participants*

Fifteen 6-month-old (5 girls, *M* = 6 months, 18 days, *SD* = 13 days) and fifteen 12-month-old (10 girls, *M* = 11 months, 19 days, *SD* = 11 days) infants participated. An additional six infants were tested but not included in the analysis due to fussiness/crying before and shortly after the beginning of the procedure or due to technical problems. The participants were recruited from a rural area in Nova Scotia, Canada, through birth announcements in the local newspaper and word of mouth. Parents gave written informed consent for their infants to participate in this study.

### *Stimuli*

The stimuli were three 50 seconds long video recordings of three male infants displaying neutral, positive or negative emotional state. In the *Neutral* video, the infant displayed neutral facial expressions and vocalizations specific for babbling, without associated emotional prosodies. In the *Positive* video, the infant displayed facial expressions specific for happiness, and laughter vocalizations [44]. In the *Negative* video, the infant manifested facial and vocal expressions specific for anger and crying [45]. In all three videos, the infants were recorded from the upper body part/torso and head, in a supine position. Infants' vocal and facial displays are socially relevant stimuli and have been previously shown to trigger emotional responses in their peers [18], [46].

Great efforts were put in creating stimuli with high ecological validity, while still controlling for other relevant factors. We were particularly interested in recording facial and vocal expressions of emotions triggered by normal events in infants' daily life, clean of any other sound noises and visual stimuli. The positive emotional state was triggered by social interaction with the caregiver; the negative emotional state was triggered by maternal separation; while the emotionally neutral babbling episode occurred in the absence of interaction with the caregiver, when the infant was laying in the crib. The resulted stimuli were cropped from longer blocks of video recordings with minimal editing in order to depict bouts of cry, laughter, and babbling with natural characteristics. Pauses inherent for both the emotional and the neutral vocalizations were present throughout the duration of stimulation. Otherwise the cry, laughter, and the neutral babbling were continuous for the entire duration of the stimulus. Although caregivers were present in the room where the stimuli have been recorded, either for recording purposes or for triggering the emotional reaction (i.e., the Positive video), they were silent and not visible for the entire duration of the stimuli. Although we have attempted to obtain recordings from same age infants, given the infrequent occurrence of emotions in recordable situations and the strict requirements for the quality of the videos, this was not possible. At the time of recording, the infant in the *Neutral* video and the one in the *Positive* video were 6-month-old, while the infant in the *Negative* video was 9-month-old. Previous newborn studies using similar paradigms suggest that vocal emotional reactions observed in a same age peer trigger higher arousal than the ones of an older infant [14], [16]. However, recent studies with infants similar in age with our participants (i.e., 3-, 6, and 9-month-old) suggest that age difference between the observer and the perceiver does not influence the responses [47]. Therefore we have concluded that the stimuli included in this study were adequate for the investigated age groups.

Parents agreed that these videos could be recorded and presented to the participants in the study. A 10 second attention grabbing video of a moving geometrical shape associated with a rattling sound was presented between conditions.

Changes in the steady state pupil diameter are primarily due to variations in the ambient light and in the light reflected by different objects in the environment or stimulus luminance [32]. Since the stimuli we have recorded included different infants on slightly different backgrounds, we have measured their indexes of photometric luminance. For each frame of the movies, we extracted the spatial average of RGB values across the image and used their weighted sum to estimate luminance (luminance  = 0.2126×*R*+0.7152×*G* +0.0722×*B* [31]). The photometric luminance averaged across all frames within a video showed that the stimuli are similar: neutral 109.56, positive 80.76, and negative 80.61. We have also used the photometric luminance frame by frame in order to explore its potential covariance with the emotional valence of the stimuli (see Results).

### *Apparatus*

Gaze and pupil size were measured using a Tobii 2150 near infrared eye tracker adjusted for bright pupil measurement, recording with a frequency of 50 Hz. A five point calibration was performed before each recording as suggested by Gredebäck, Johnson, and von Hofsten [48]. The 21” TFT eye tracker monitor was placed against a beige curtain and no other stimuli were present that could distract the participant's attention.

A gamma correction procedure was used to ensure that luminance at the viewing location was linearly related to the RGB values. Luminance measurements were made using a Reed Instruments lux meter and the experimental monitor TFT eye tracker monitor placed against the beige background. Single measurements recorded during every 5 s of each stimulus presentation showed that illuminance was constant with 15.10 (±.10) Lux for the neutral, 15.30 (±.10) Lux for the positive and 15.40 (±.10) Lux for the negative video. The light in the experimental room was kept constant across participants at 13.10 Lux.

### *Procedure*

After a period of adjustment to the testing environment, infants were seated in an age appropriate car seat. The caregiver was seated behind the participant, but within a few centimeters distance. When ready, the infant seat was rolled in front of a monitor and the position adjusted for good gaze tracking. After calibration, the procedure began with the presentation of the attention grabber. For half of the participants the procedure continued with the presentation of the neutral condition followed by the positive one. For the other half, the order of presentation for the neutral and positive condition was reversed. The negative stimulus was always presented last as previous studies report that such stimuli induce strong and persistent negative emotional reactions in infants [12], which could have a carryover effect and could lead to procedure termination. Each of the 50 seconds video stimuli was presented once.

### *Data reduction*

#### Looking time

In order to analyze the pattern of visual fixation, we defined two areas of interest for each stimulus. The first area of interest covered the face of the infant depicted in the video and an area of approximately 20 pixels surrounding it. The second area of interest covered the rest of the image. From the total duration of the infant looking time to the stimulus, the proportion of gazing to each area of interest was then computed.

#### Pupil data

Initially, samples where the pupil was obscured by blinking were identified and removed. Then a digital low-pass filter with a cut frequency ratio of 12.5 was applied in order to remove sudden brief increases and decreases in pupil diameter that normally occur and are considered to be artifacts [33]. In order to avoid phase drift, the filter was applied a second time, backwards. Missing samples were interpolated linearly from the average of the last three samples before the break to the average of the first three samples after the break. Where data from one eye was missing, pupil data from the other eye was used for interpolation. Pupil data from both eyes at each sample was averaged and this value was used for further analyses.

An initial inspection of the data revealed a trend for a significant data loss (more than 50%) during the second half of the stimulus presentation, mainly due to increased head motion and long gazes away from the monitor. As a result, the data only for the first 25 s of stimulus presentation was analyzed.

**Figure 1:**
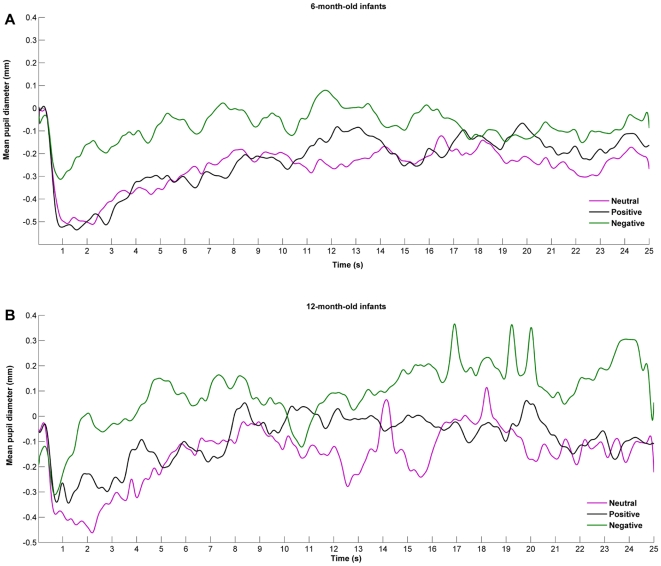
Mean functional change in pupil diameter for each type of stimulus. After filtration and interpolation, the pupil measurements from both eyes were averaged. Baseline-correction was performed by subtracting the mean pupil diameter from the last one second of the preceding inter-trial from each data point recorded during the stimulus presentation. The change in pupil size from baseline for the neutral (purple line), positive (black line), and negative (green line) stimulus is presented separately for 6- (A) and 12-month-old infants (B).

Baseline-correction was performed by subtracting the mean pupil diameter from the last one second of the preceding inter-trial from each data point recorded during the stimulus presentation. This choice of baseline correction was motivated primarily by the emotional nature of the stimuli and the repeated measures design. In this particular case there is the risk that residual stimulation persists after stimulus offset, altering the ‘resting’ state on which the following stimulus occurs. The use of a baseline immediately before the stimulus onset allows correction for these possible effects [32], [34]. Important data loss during baseline occurred due to participant inattention, which limited the baseline duration option to 1 s. Previously, this baseline length has been proved adequate [31], [33]. Two 12-month-old participants did not have sufficient data during one of the baselines, therefore they were removed from further analysis at this point. Figure 1 shows the baseline corrected pupil diameter for each condition, separately for each age group. During the first two seconds after stimulus onset, as expected, the light reflex occurred due to the change in luminance from interstimulus to stimulus presentation. As a consequence of this, further analysis of the condition effects excluded this time window [35].

Further on, given that pupil diameter varies as a function of time, we used functional data analysis to analyze possible differences between conditions. We fitted the baseline-corrected data by using B-splines functions of order 4 with 28 bases. This type of polynomial function was shown to be adequate for analyzing changes in pupil diameter in infants (for further details on functional data analysis and its application to pupillary reactivity, see [31], [49]). For each participant, for each condition, the total number of data points was converted into one functional expression which was further used for analysis. By using the functional expression of the variation in pupil size along the duration of the stimulus, we also addressed the issue of performing a large number of comparisons that would be otherwise required for covering all data points.

## Results

### *Looking time*

**Figure 2:**
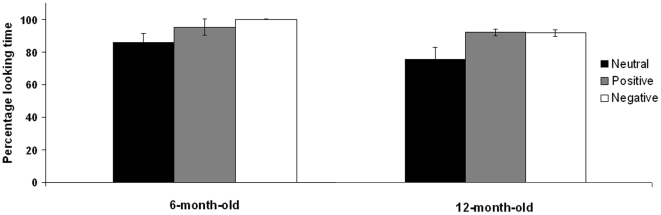
Looking time to the peer's face. Looking time to the peers' faces during the neutral (black), positive (gray), and negative (white) emotional display was computed as percentage from the total looking time towards the stimulus. Error bars represent *SE*s.

On average, for all stimuli, participants from both age groups spent most of their looking time gazing at the infants' face rather than at the rest of the image (neutral *t*(28) = 6.477, *p* = .001); positive (*t*(30) = 17.041, *p* = .001), negative (*t*(28) = 23.466, *p* = .001); Figure 2). A mixed ANOVA 3 (emotion: negative, positive, neutral) X 2 (age: 6-, 12-month-old) showed a significant effect of emotion (*F*(2, 28) = 9.294, *p* = .002, *η^2^* = .263, Greenhouse-Geisser correction), but no significant effect of age (*F*(1, 28) = 1.630, *p* = .213, η*^2^* = .059) or age and emotion interaction (*F*(2, 28) = .453, *p* = .638, *η^2^* = .042). Infants spent a larger proportion of time gazing at the laughing (*t*(28) = −3.005, *p* = .006) and at the crying infant face (*t*(27) = −3.942, *p* = .001) than at the emotionally neutral one. However, no significant differences were found between the percentage of time spent looking at the crying infant face than at the laughing face (*t*(28) = −.867, *p* = .394).

### *Pupil size: Light effects*

For both age groups the reflex pupil constriction to the onset of the stimulus was recorded as expected within the first 2 seconds.

**Figure 3:**
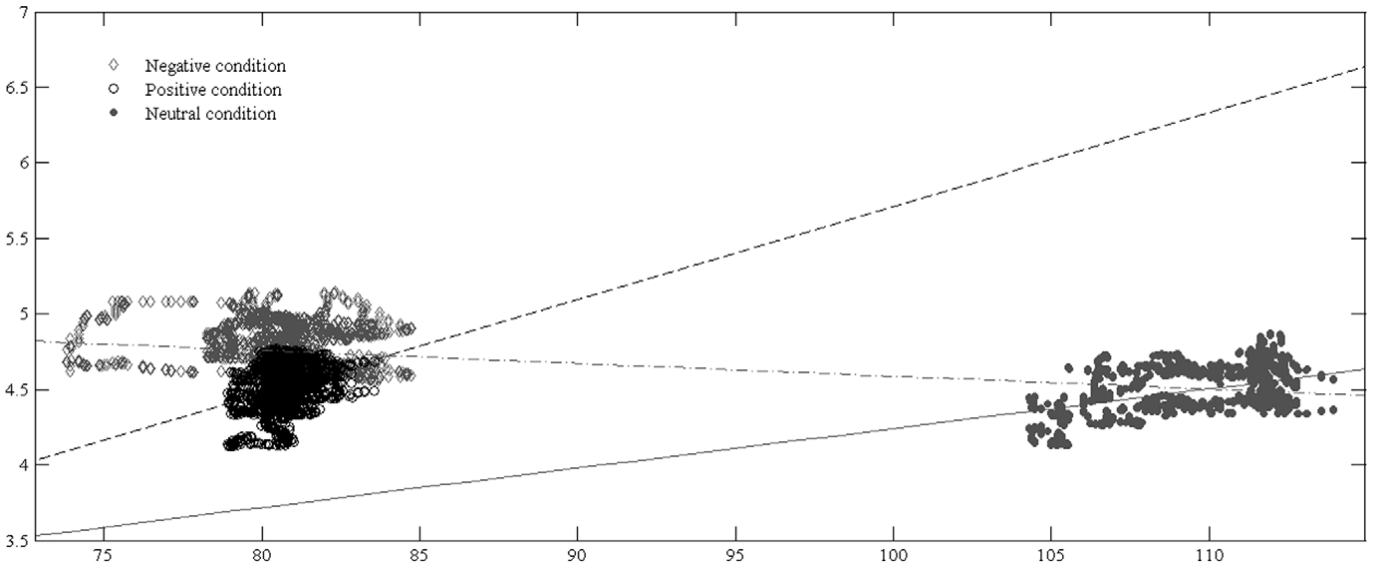
The effect of stimulus luminance on pupil dilation. We explored whether variations in stimulus luminance across video frames have a significant effect on the recorded changes in pupil size. It was predicted that as stimulus luminance increases, an adjustment reaction with decreasing pupil size would be recorded. It was found that the emotional valence of the stimuli explained a big proportion of the variation, *partial* *η^2^* = .32, while variations in luminance across the movies had an extremely small effect, *partial* *η^2^* = .04. The relation between the effect of luminance on pupil size differed across emotions (*F*(2, 7500) = 217.77, *p*<.001). When controlling for other sources of variations between videos [69], the relation between luminance and pupil size is positive (.42), and this could be due to the overall tendency of the pupil to increase with stimulus presentation. Illuminance measured at the level of the eye during the presentation of each video was within a range not expected to yield significant differences in pupil size (15.0±0.5 Lux) [50]. All this information suggests that the observed differences in luminance and illuminance between stimuli could not produce the differences in pupil size related to the emotional valence.

The effect of spatial average of RGB values for each frame of the movies as a covariate for the emotional condition for the baseline corrected data before the use of functional analysis was analyzed with ANCOVA. Variations in luminance across the movies although significantly related to pupil diameter, *F*(1, 7500) = 310.52, *p*<.001, had an extremely small effect, *partial* *η^2^* = .04. The effect of the emotion condition after controlling for the effect of luminance was significant *F*(2, 7500) = 1773.85, *p*<.001 and explaining a big proportion of the variation, *partial* *η^2^* = .32. Importantly, the relation between the effect of luminance on pupil size differed across emotions (*F*(2, 7500) = 217.77, *p*<.001; Figure 3) with a trend inconsistent with hypothesis that the observed changes are due to luminance. Importantly, the illuminance during stimulus presentation was within a range not expected to lead to differences in pupil dilation (15.0±0.5 Lux) [50]. Therefore, although differences in luminance between stimuli have a small influence, an important part of the variation in pupil size is related to differences in the emotional content.

### *Pupil size: Emotion effects*

First, we have analyzed the average changes in pupil size for the entire duration of stimulus presentation after the light reflex (2–25 s). A mixed 3 (condition) by 2 (age) ANOVA shows a significant effect of condition (*F*(2,28) = 11.970, *p* = .001, η*^2^* = .358), but no effect of age (*F*(1,28) = .869, *p* = .360, *η^2^* = .032) or interaction (*F*(2,28) = .095, *p* = .910, *η^2^* = .010). Planned repeated measures comparisons for the entire sample showed that another infant's cry triggered larger changes in pupil dilation compared to the neutral vocalizations (*t*(27) = 3.879, *p* = .001) and laughter (*t*(27) = 3.810, *p* = .001). In average, no significant differences were recorded between laughter and emotionally neutral vocalizations (*t*(27) = .754, *p* = .458).

**Figure 4:**
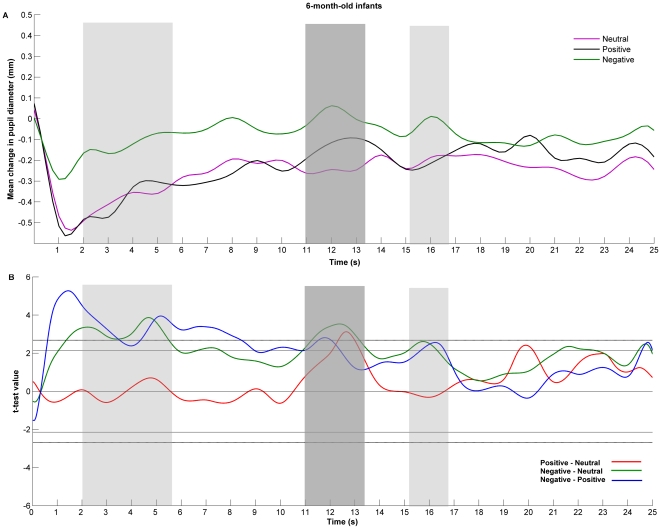
Six-month-old infants' pupil size changes in response to others' affective displays. In Panel A, functional expression of changes in the 6-month-olds baseline corrected pupil size in response to others' neutral (purple line), positive (black line), and negative (green line) emotional displays. Panel B shows the value of t statistic as a function of time for each pair of stimuli comparison: positive vs. neutral (red line), negative vs. neutral (green line), and negative vs. positive (blue line). The one-tailed critical values of t are represented by the vertical lines with (dotted) and without (solid) Bonferroni correction for three comparisons. The difference is considered to be significant when this line is reached or surpassed. Marked with dark gray are those time windows in which both emotional conditions triggered larger pupil diameters than the neutral one, and the hypothesized negativity bias occurred. Marked with light gray are those time windows where negative emotions triggered larger pupil diameter compared to the emotionally neutral state. (Note that the shaded areas are for illustrative purposes only, marking the time window in which the effect occurred and not the effect duration.)

**Figure 5:**
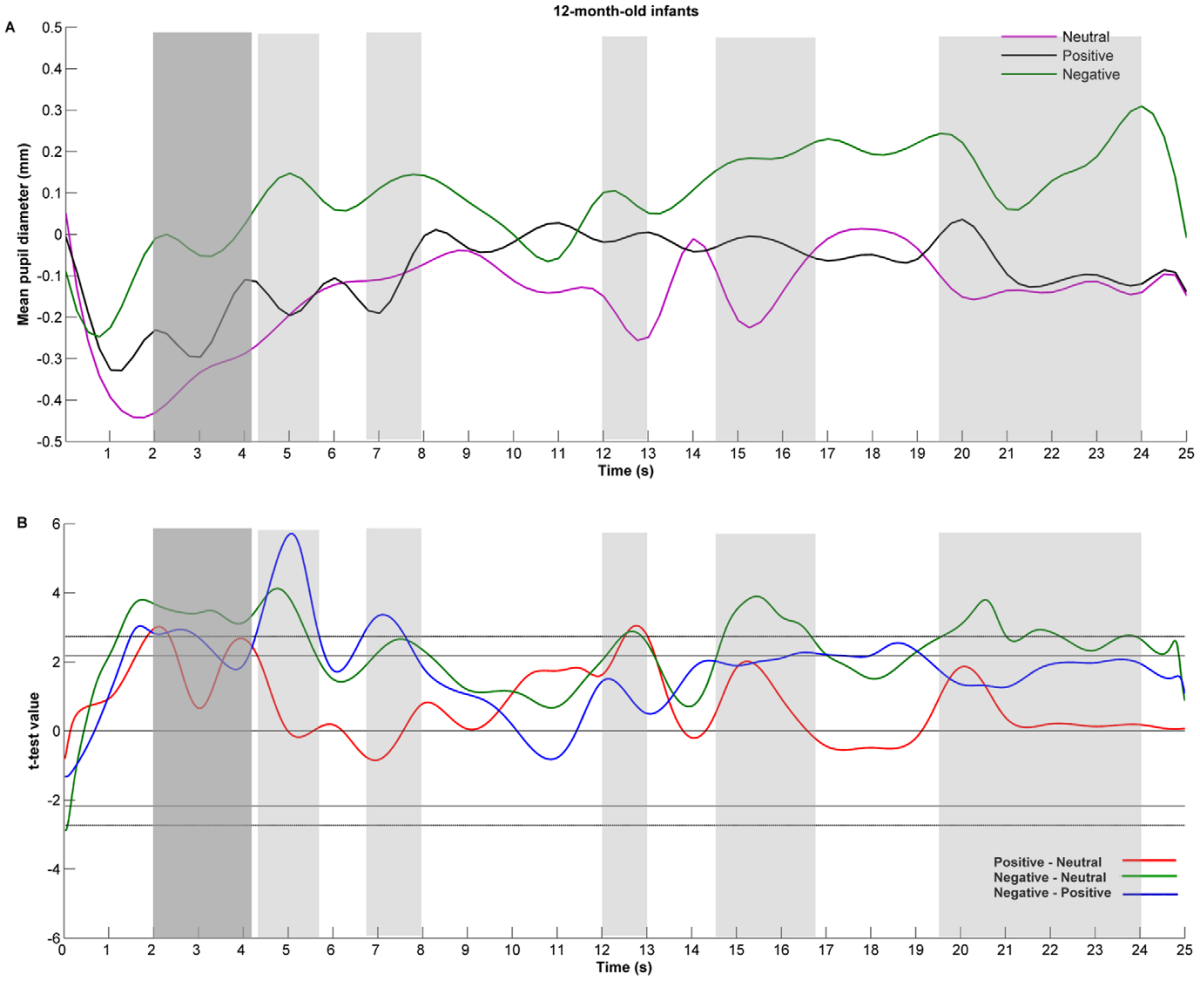
Twelve-month-old infants' pupil size changes in response to others' affective displays. In Panel A, functional expression of changes in the 12-month-olds baseline corrected pupil size in response to others' neutral (purple line), positive (black line), and negative (green line) emotional displays. Panel B shows the value of t statistic as a function of time for each pair of stimuli comparison: positive vs. neutral (red line), negative vs. neutral (green line), and negative vs. positive (blue line). The one-tailed critical values of t are represented by the vertical lines with (dotted) and without (solid) Bonferroni correction for three comparisons. The difference is considered to be significant when this line is reached or surpassed. Marked with dark gray are those time windows in which both emotional conditions triggered larger pupil diameters than the neutral one, and the hypothesized negativity bias occurred. Marked with light gray are those time windows where negative emotions triggered larger pupil diameter compared to the emotionally neutral state. (Note that the shaded areas are for illustrative purposes only, marking the time window in which the effect occurred and not the effect duration.)

However, the average change in pupil size for the entire duration of the stimulus allows only a global analysis of the responses. In order to look at the time course of changes in pupil size as a function of the stimuli emotional valence, spline functions were created for each participant for each condition and then averaged across the entire sample within each age group (see Figure 4 and 5, panel A, for mean functional pupil diameter for each stimulus, separately for each age group).

In order to analyze whether the pupil diameter in response to peers' emotions is higher than for peer's emotionally neutral state, and whether negative emotions triggered a larger pupil diameter than the positive one, we performed planned repeated measures comparisons (1-tailed) for each age group. Figures 4 and 5 (panel B) show the results of *t-*test as a function of time, with Bonferroni correction for three comparisons. In 6-month-old infants, after 2 seconds from onset, significant differences occurred in pupil size changes between negative and neutral, and between negative and positive conditions, that lasted for almost four seconds. These differences tended to occur again around the 16th second of stimulus presentation. In the time interval 11 to 13.5 seconds, we recorded changes in pupil diameter that were significantly different between all conditions.

In contrast, 12-month-old infants tended to record much earlier a larger pupil diameter for the emotional conditions compared to the neutral one, paralleled by a differentiation between the emotional conditions (within the 2 to 4 seconds interval after stimulus onset). Increased pupil dilation in response to the crying peer compared to the neutral vocalizations and laughter lasted for a major part of the 2 to 8 seconds time interval. The larger pupil diameters for the emotional versus neutral conditions occurred again in the 12 to 13 seconds time interval, while the negative emotion continued to elicit such differences for almost the rest of the stimulation.

Taken together, both analyses of changes in pupil size suggest that the negative emotional stimulus induces larger pupil sizes than the neutral and the positive ones. However, only the functional data analysis revealed differences between the positive and the neutral stimulus, and the time related aspect of these effects.

No significant correlations were recorded between looking time measures and changes in pupil size (*r*s<−.36, *p*s>.05).

To summarize, the analysis of looking behaviour shows that both 6- and 12-month-old infants gazed for an equal amount of time at the face of their peer during the emotional conditions. More importantly, the analysis of pupil size suggests that for both ages, pupil diameter differed significantly between conditions. Watching other's negative emotions led to larger pupil sizes when compared to both positive and neutral emotional displays in 6- and 12-month-old infants. Importantly, there were several time windows where attending to other's positive emotions tended to trigger larger pupil diameters than the emotionally neutral states. For older infants such a differentiation tended to occur earlier during the stimulus presentation than for the younger ones.

## Discussion

Results of this study show that observing peers' emotions induces in infants an increase in pupil size. These effects varied with the valence of emotion. Other's distress triggered larger pupil size for both 6- and 12-month-old infants for extended periods of time. Perceiving other's happiness induced larger pupil diameters as compared to the emotionally neutral state but for shorter time intervals. An asymmetry in responses, with larger pupil sizes for another's distress compared to another's happiness, was recorded for both age groups, but during different time windows. The socio-emotional relevant aspects of the stimuli (i.e., the peer's face) were the main focus of the infant's attention at both ages. However, infants looked equally long to both positive and negative emotions during stimulus presentation. Together with previous findings showing the validity of changes in pupil size as a measure of emotional arousal [35], the current study provides for the first time evidence to suggest that infants respond with arousal to others positive and negative emotions.

It has been suggested that others' negative emotions are more likely to lead to shared affect in the observer than the positive ones [24], and that this bias towards negativity is not present from birth, but rather develops towards the end of the first year of life [20]. Our results support only partially these hypotheses. Observing others' negative emotions induces autonomic arousal in both younger and older infants as has been previously shown [9], [12], [16], [18]. Peer's positive emotions triggered as well larger pupil diameter compared to the emotionally neutral states, although during limited time windows. For those time intervals where positive vs. neutral differentiation in pupil diameter was observed, we also found the hypothesized asymmetry with the negative emotions. Thus, while for extended periods of time the perception of a crying peer triggered higher arousal than the laughing one, the latter was only at times different than perceiving a peer in an emotionally neutral state. Importantly, this fine differentiation in results was possible due to the use of functional data analysis with B-splines [31].

Empathy is a complex emotional state triggered by, and similar to, the emotion experienced by another person, and it encompasses both responses of negative and positive affect. Evidence has only recently emerged to show that 6-year-old children experience empathic happiness when they see a happy person, demonstrating that it is not only distress that can trigger such reactions [51]. In our study children already in their first year of life reacted with autonomous arousal to others' happiness.

Variations in sympathetically driven pupil diameter have been consistently found in response to emotional stimuli. In adults, presentation of emotionally positive and negative sounds and pictures lead to larger pupil diameters than emotionally neutral stimuli, as early as two to six seconds after the stimulus onset [35], [36]. In children, however, words with negative emotional load lead to larger pupil sizes than those with positive valence [42]. In the current study we have shown that in infants, perception of facial and vocal expressions of distress lead to larger pupil diameter than perception of positive emotional displays. This could be due to the increased allocation of attention recorded at the level of brain activity for negative emotional information [25], [52]. Whereas both laughing and crying are important vocal expressions of emotional states [53]–[55], the sound of crying and negative emotional prosodies may have a higher social relevance because they signal threat. An increased allocation of attention and general arousal to these stimuli has a potentially adaptive value in this scenario. However, it has been found that in 7-month-old infants speech positive emotional prosodies lead to increased activation not just in the voice sensitive superior temporal cortex, but also in the inferior frontal cortex known to be involved in more detailed speech processing. This is probably because of the association between positive emotional prosodies and infant-directed speech [56]. Our findings seem also consistent with this interpretation, suggesting that although positive emotional vocalizations are processed, this is not necessarily associated with increased emotional arousal. More importantly, as has been previously shown, there was not a significant relation between these two measurements, which could suggest that they index different processes [31], [43]. Our design is suitable for recording variations in pupil size, but it is not perfectly adequate for analyzing and interpreting looking behaviour in terms of discriminatory abilities, where visual habituation designs would be more appropriate [57]. However, it does suggest that both 6- and 12-month-old infants visually processed the positive emotional stimulus for a longer duration than the neutral one. Such findings further demonstrate the validity of pupillary recordings as a measure of autonomous sympathetic arousal for studying infants' and children's differential responses to emotionally loaded stimuli [35]. We also know that changes in pupil size are sensitive to the cognitive processing demands of the stimuli [30], [31], [33], hence differentiation between pupil responses to these categories of stimuli in the developing populations would be of great value. As previously shown [35], [37], the recording of concurrent electrophysiological measurements could be the strategy of choice for further investigations.

In Haviland and Lelwica's study [9], 10-week-old infants responded with matched facial expressions of happiness to their mothers' joy that was expressed at the level of the face and prosody. In our study, although after a longer delay, 6-month-old infants show larger pupil diameters to happiness than to emotionally neutral expression. One possible explanation for this very late and time limited effect could be related to the identity of the social agent presented in the video and the quality of vocal expression of emotion. While the simple joyful presence of the mother could be very fast perceived as an initiation of social interaction that consequently triggers positive emotional responses, it is probably less likely for younger infants to respond with arousal to the joyful presence of a peer which is not a frequent social agent in their environment. Importantly, while in previous studies, vocal expression of happiness was embedded in verbal utterances [9], [25], [58], in our current study they were embedded within specific stereotyped laughing vocalizations [59]. While little is known about infant processing of laughter as a vocal expression of joy and the equivalent expression of crying as an expression of distress [53], the results of our study suggest that after the age of 6 months, children begin to manifest arousal when perceiving their peer's joyful face and laughter.

Non-linguistic emotional vocalizations like crying and laughter represent essential means of expressing emotions, complementary to facial expressions and verbal related prosodies [53], [60]. While cry vocalizations are already present at birth and reach stable auditory properties by the age of 2-months [61], laughs occur mostly starting at 4-months of age, when they become significantly associated with positive emotions [62]. When adults are presented with positive and negative non-linguistic emotional vocalizations, increased brain activity is recorded at the level of those areas involved in processing complex acoustic information in human voices (the superior temporal sulcus and the primary auditory cortex, superior temporal gyrus, and the amygdala) [63]. At the age of 7-months, but not earlier, the pattern of neural activation associated with processing emotional and non-emotional verbal utterances resembles the one recorded in adults [64], [56]. The current study along with previous findings indicates that newborns and infants manifest autonomous arousal responses to negative emotional non-linguistic vocalizations either associated or not with the corresponding facial expression, suggesting that specific processing mechanisms are already functioning [15], [16], [18]. It remains to be established whether the neural correlates of processing emotional non-linguistic vocalizations develop concomitantly with those for verbal prosodies, and whether they are simultaneous for positive and negative valence.

By using nonverbal emotional vocalizations and the associated facial expressions displayed by infants, we have reduced the possibility that the reactions recorded in participants are the result of social interaction rather than a response to other's affect. However, one might argue that the acoustic characteristics of the cry, laughter and babbling are to explain the observed differences in pupil diameter. Previous investigations have shown that white noise can induce an increase in arousal. However, a cry sound with the same intensity triggers significantly higher levels of arousal [16]. In terms of changes in pupil diameter, loud sounds without emotional valence have been found to trigger rather small increases in pupil diameter of about.04 mm that habituate with repeated presentation [65]. Our results suggest that, at least for 12-month-old-infants, cry sounds which are the most intense lead to increases in pupil diameter from.10 to.30 mm, which rather increase with continuous presentation. This suggests, that the observed changes are less likely to be due to the sound intensity per se, and more to the social information it provides. Differences in the onset of vocalizations bouts may also be relevant for the recorded differences in pupil diameter. If this would be the case, then similar profiles of changes in pupil diameter would be recorded for both age groups [66]. For most of the stimulus duration these predictions cannot be confirmed. However, a more firm dismissal of such alternative interpretation could be facilitated, for example, by a multiple trial design, in which a variety of nonverbal vocalizations stimuli are used for each emotion. This would allow us to understand how infant pupillary responses change as a function of sound characteristics, and whether these interact with the associated emotional information.

There are several limitations of the study that deserve to be addressed in future research. We have recorded differences in the initial pupil constriction to the change in the visual display, and they seem to be different across age groups. One possible explanation could be the difference in the visual characteristics of the stimuli other than luminance, like color, spatial frequency, and motion [66]. Although difficult to control for when using ecologically valid video recorded stimuli, future studies may address this issue by employing static visual display of emotions associated with vocal expressions, and by including different age groups. Also, interindividual and age related differences in reactivity and inhibition may provide additional information. In adults, interindividual differences in both impulsivity and trait anxiety have been found to influence the light reflex amplitude, independent of the pupil diameter at rest [65], [67]. In our study, information about infants temperamental characteristics were not recorded, thus it is difficult to estimate whether these factors explain the observed differences in the pupil light reflex. Also, although we have tried to limit the influence of other social characteristics of the stimuli (e.g., initiatives of social interaction), these may still be present, especially in older infants who have more advance social cognition skills.

Emotional resonance to others' emotional experiences is the defining characteristic of empathy. Recent findings have shown that throughout the first year of life, infants react with negative emotions to their peers' negative affect. These responses are regulated through age specific mechanisms and, from about the age of 9 months, also associated with bodily self-awareness [12], [47], key processes in the development of empathy [68]. Our current results add to the knowledge by showing that 6- and 12-month-old infants manifest increased arousal not just to other's negative emotions, but also to others' positive affect expressed in face and voice. This is of high relevance for understanding the early development of empathy, with further implications for investigating the emergence of empathic concern and happiness in early childhood.

## References

[1] Ludemann PM (1991). Generalized discrimination of positive facial expressions by 7- to 10-month-old infants.. Child Development.

[2] Nelson CA (1987). The recognition of facial expressions in the first year of life: Mechanisms of development.. Child Development.

[3] Baldwin DA, Moses LJ (1996). The ontogeny of social information gathering.. Child Development.

[4] Eisenberg N, Shea CL, Carlo G, Knight G, Kurtines W, Gewirtz J (1991). Empathy related responding and cognition: A “chicken and the egg” dilemma.. Handbook of Moral Behavior and Development, Vol2: Research.

[5] Cappella JN (1993). The Facial Feedback Hypothesis in Human Interaction.. Journal of Language and Social Psychology.

[6] Decety J (2011). Dissecting the Neural Mechanisms Mediating Empathy.. Emotion Review.

[7] Lipps T (1907). Das Wissen von fremden Ichen.. Psychologische Untersuchungen 1.

[8] Blair RJR (2011). Should Affective Arousal be Grounded in Perception-Action Coupling?. Emotion Review.

[9] Haviland JM, Lelwica M (1987). The induced affect response: 10-week-old infants' responses to three emotion expressions.. Developmental Psychology.

[10] Hess U, Blairy S (2001). Facial mimicry and emotional contagion to dynamic emotional facial expressions and their influence on decoding accuracy.. International Journal of Psychophysiology.

[11] Jones SS (2009). The development of imitation in infancy.. Philosophical Transactions of the Royal Society Biology.

[12] Geangu E, Benga O, Stahl D, Striano T (2010). Contagious crying beyond the first days of life.. Infant Behavior and Development.

[13] Dondi M, Simion F, Caltran G (1999). Can newborns discriminate between their own cry and the cry of another newborn infant?. Developmental Psychology.

[14] Martin G, Clark R (1982). Distress crying in neonates: Species and peer specificity.. Developmental Psychology.

[15] Sagi A, Hoffman ML (1976). Empathic Distress in the Newborn.. Developmental Psychology.

[16] Simner ML (1971). Newborn's response to the cry of another infant.. Developmental Psychology.

[17] Field T, Diego M, Hernandez-Reif M, Fernandez M (2007). Depressed Mothers' Newborns Show Less Discrimination of Other Newborns' Cry Sounds.. Infant Behavior and Development.

[18] Hay DF, Nash A, Pedersen J (1981). Responses of six-month-olds to the distress of their peers.. Child Development.

[19] Serrano JM, Iglesias J, Loeches A (1995). Infants' responses to adult static facial expressions.. Infant Behavior and Development.

[20] Vaish A, Grossmann T, Woodward A (2008). Not all emotions are created equal: The negativity bias in social-emotional development.. Psychological Bulletin.

[21] Mumme D, Fernald A (2003). The infant as onlookers: Learning from emotional reactions observed in a television scenario.. Child Development.

[22] Mumme DL, Fernald A, Herrera C (1996). Infants' responses to facial and vocal emotional signals in a social referencing paradigm.. Child Development.

[23] de Gelder B, Snyder J, Greve D, Gerard G, Hadjikhani (2004). Fear fosters flight: A mechanism for fear contagion when perceiving emotion expressed by a whole body.. Proceedings of the National Academy of Sciences.

[24] Rozin P, Royzman EB (2001). Negativity bias, negativity dominance, and contagion.. Personality and Social Psychology Review.

[25] Grossmann T, Striano T, Friederici AD (2005). Infants' electric brain responses to emotional prosody.. NeuroReport.

[26] Grossmann T, Striano T, Friederici AD (2007). Developmental changes in infants' processing of happy and angry facial expressions: A neurobehavioral study.. Brain & Cognition.

[27] Hornick R, Risenhoover N, Gunnar M (1987). The effects of maternal positive, neutral, and negative affective communications in infant responses to new toys.. Child Development.

[28] Baumeister RF, Bratslavsky E, Finkenauer C, Vohs KD (2001). Bad is stronger than good.. Review of General Psychology.

[29] Camras LA, Shutter JM (2010). Emotional facial expressions in infancy.. Emotion Review.

[30] Gredebäck G, Melinder A (2010). Infants' understanding of everyday social interactions: a dual process account.. Cognition.

[31] Jackson I, Sirois S (2009). Infant cognition: going full factorial with pupil dilation.. Developmental Science.

[32] Lowenstein O, Loewenfeld IE, Davidson H (1962). The pupil.. The Eye Muscular Mechanisms (Vol3).

[33] Beatty J, Lucero-Wagoner B, Caccioppo J, Tassinary LG, Berntson, G (2000). The pupillary system.. The Handbook of Psychophysiology.

[34] Hess EH, Polt JM (1960). Pupil size as related to interest value of visual stimuli.. Science.

[35] Bradley MM, Miccoli L, Escrig MA, Lang PJ (2008). The pupil as a measure of emotional arousal and autonomic activation.. Psychophysiology.

[36] Partala T, Surakka V (2003). Pupil size variation as an indication of affective processing.. International Journal of Human-Computer Studies.

[37] Critchley HD, Tang J, Glaser D, Butterworth B, Dolan RJ (2005). Anterior cingulate activity during error and autonomic response.. Neuroimage.

[38] Bitsios P, Szabadi E, Bradshaw CM (1998). Sensitivity of the fear-inhibited light reflex to diazepam.. Psychopharmacology.

[39] Siegle GJ, Steinhauer SR, Carter CS, Ramel W, Thase ME (2003). Do the seconds turn into hours? Relationships between sustained pupil dilation in response to emotional information and self-reported rumination.. Cognitive Therapy Research.

[40] Steinhauer SR, Hakerem G (1992). The pupillary response in cognitive psychophysiology and schizophrenia. In: Friedman D, Bruder G editors. Psychophysiology and experimental psychopathology: A tribute to Samuel Sutton.. Annals of the New York Academy of Sciences.

[41] Fitzgerald HE (1968). Autonomic pupillary reflex activity during early infancy and its relation to social and non-social visual stimuli.. Journal of Experimental Child Psychology.

[42] Silk JS, Dahl RE, Ryan ND, Forbes EE, Axelson DA (2007). Pupillary reactivity to emotional information in child and adolescent depression: links to clinical and ecological measures.. American Journal of Psychiatry.

[43] Decety J, Michalska KJ, Kinzler KD (2011). The contribution of emotion and cognition to moral sensitivity: a neurodevelopmental study.. Cerebral Cortex.

[44] Messinger DS, Fogel A, Dickson KL (2001). All smiles are positive, but some smiles are more positive than others.. Developmental Psychology.

[45] Sullivan MW, Lewis M (2003). Emotional expressions of young infants and children – a practitioner's primer.. Infants and young children.

[46] Legerstee M, Anderson D, Schaffer A (1998). Five- and eight-month-old infants recognize their faces and voices as familiar and social stimuli.. Child Development.

[47] Geangu E, Benga O, Stahl D, Striano T (2011). Individual Differences in Infants' Emotional Resonance to a Peer in Distress: Self–Other Awareness and Emotion Regulation.. Social Development.

[48] Gredebäck G, Johnson S, von Hofsten C (2010). Eye tracking in infancy research.. Developmental Neuropsychology.

[49] Ramsay JO, Silverman, BW (2005). Functional Data Analysis..

[50] MacLachlan C, Howland HC (2002). Normal values and standard deviations for pupil diameter and interpupillary distance in subjects aged 1 month to 19 years.. Ophthalmological Physiology and Optometry.

[51] Light SN, Coan JA, Zahn-Waxler C, Frye C, Goldsmith HH (2009). Empathy is associated with dynamic change in prefrontal brain electrical activity during positive emotion in children.. Child Development.

[52] Vuilleumier P (2005). How brains beware: neural mechanisms of emotional attention.. Trends in Cognitive Sciences.

[53] Davila Ross M, Owren MJ, Zimmerman E (2009). Reconstructing the evolution of laughter.. Current Biology.

[54] Green JA, Gustafson GE, Irwin JR, Kalinowski LL, Wood RM (1995). Infant crying: Acoustics, perception and communication.. Early Development and Parenting.

[55] Gustafson G, Wood R, Green J, Barr R, Hopkins B, Green J (2000). Can we hear the causes of infants' crying?. Crying as a sign, a signal, and a symptom.

[56] Grossmann T, Oberecker R, Koch SP, Friederici AD (2010). Developmental origins of voice processing in the human brain.. Neuron.

[57] Nelson CA, Morse PA, Leavitt LA (1979). Recognition of facial expressions by seven-month-old infants.. Child Development,.

[58] Walker-Andrews AS, Lennon EM (1991). Infants' discrimination of vocal expressions: Contributions of auditory and visual information.. Infant Behavior and Development.

[59] Provine RR, Yong YL (1991). Laughter: A stereotyped human vocalization.. Ethology.

[60] Scherer KR, Darby J (1981). Speech and emotional states.. Speech evaluation in psychiatry.

[61] Wermke K, Mende W, Manfredi C, Bruscaglioni P (2002). Developmental aspects of infant's cry melody and formants.. Medical Engineering Physics.

[62] Scheiner E, Hammerschmidt K, Jürgens U, Zwirner P (2002). Acoustic analyses of developmental changes and emotional expression in the preverbal vocalizations of infants.. Journal of Voice.

[63] Fecteau S, Belin P, Joanette Y, Armony JL (2007). Amygdala responses to nonlinguistic emotional vocalizations.. NeuroImage.

[64] Belin P, Grosbras M-H (2010). Before speech: cerebral voice processing in infants Neuron.

[65] Frith CD (1981). The effects of sound on pupil size and the pupil light reflex.. Personality and Individual Differences.

[66] Barbur JL, Chalupa LM, Werner JS (2004). Learning from the pupil - studies of basic mechanisms and clinical applications.. The Visual Neurosciences.

[67] Bitsios P, Szabadi E, Bradshaw CM (2002). Relationship of the "fear-inhibited light reflex" to the level of state/trait anxiety in healthy subjects.. International Journal of Psychophysiology.

[68] Hoffman ML (2000). Empathy and moral development..

[69] Bland JM, Altman DG (1995). Calculating correlation coefficients with repeated observations: Part 1 – correlation within subjects.. BMJ.

